# Local clothing thermal properties of typical office ensembles under realistic static and dynamic conditions

**DOI:** 10.1007/s00484-018-1625-0

**Published:** 2018-10-29

**Authors:** Stephanie Veselá, Agnes Psikuta, Arjan J. H. Frijns

**Affiliations:** 1 Department of Mechanical Engineering, Eindhoven University of Technology, P.O. Box 513, 5600 MB Eindhoven, The Netherlands; 2 Empa - Swiss Federal Laboratories for Materials Science and Technology, Lerchenfeldstr. 5, 9014 St. Gallen, Switzerland

**Keywords:** Local clothing insulation, Local area factor, Thermal modeling, Office clothing ensembles

## Abstract

An accurate local thermal sensation model is indispensable for the effective development of personalized conditioning systems in office environments. The output of such a model relies on the accurate prediction of local skin temperatures, which in turn depend on reliable input data of the local clothing thermal resistance and clothing area factor. However, for typical office clothing ensembles, only few local datasets are available in the literature. In this study, the dry thermal resistance was measured for 23 typical office clothing ensembles according to EN-ISO 15831 on a sweating agile manikin. For 6 ensembles, the effects of different air speeds and body movement were also included. Local clothing area factors were estimated based on 3D scans. Local differences can be found between the measured local insulation values and local area factors of this study and the data of other studies. These differences are likely due to the garment fit on the manikin and reveal the necessity of reporting clothing fit parameters (e.g., ease allowance) in the publications. The increased air speed and added body movement mostly decreased the local clothing insulation. However, the reduction is different for all body parts, and therefore cannot be generalized. This study also provides a correlation between the local clothing insulation and the ease allowance for body parts covered with a single layer of clothing. In conclusion, the need for well-documented measurements is emphasized to get reproducible results and to choose accurate clothing parameters for thermo-physiological and thermal sensation modeling.

**Electronic supplementary material:**

The online version of this article (10.1007/s00484-018-1625-0) contains supplementary material, which is available to authorized users.

## Introduction

Most adults spend the major part of the day at work, typically in an office building. To enable workers at office buildings to perform at their best and stay healthy, it is necessary that the indoor environment meets their individual needs (Seppänen et al. 2006; Urlaub et al. 2013). However, office buildings also have to be energy efficient to adhere to modern standards. Hence, researchers and building engineers aim to design the buildings’ heating, ventilation, and air conditioning systems to be energy efficient, while also providing a thermally comfortable environment to all occupants. To achieve this goal, personalized heating and cooling systems are being developed (Arens et al. 1991; Melikov et al. 1994; Foda and Sirén 2012; Veselý and Zeiler 2014; Parkinson et al. 2015). To test the thermal comfort provided by these systems, a large number of human subjects are usually required, which increases the studies’ cost and length. This situation could be improved by using local thermal sensation and coupled thermal comfort models for preselecting promising designs. To achieve a high predictability, these models require reliable input data of the local clothing thermal resistance and clothing area factor. However, Veselá et al. (2017) show that the available data is limited for typical office clothing ensembles. Furthermore, few studies were performed on the local effect of increased air speeds and body movement on the dry thermal resistance.

Studies published on local clothing insulation values are for example Lee et al. (2013), Lu et al. (2015), Nelson et al. (2005), and Havenith et al. (2012). In Lee et al. (2013), measurements were carried out on a thermal manikin seated on a chair with different clothing sets. Their study contained a large variety of ensembles, but the effect of air speed and body movement were not included. Lu et al. (2015) studied the effect of air speed and body movement for dry local clothing insulation, but only two clothing ensembles are usable for office settings. A different approach is found in Nelson et al. (2005), where local insulation values were recalculated from the global values published by McCullough et al. (1985, 1989). Their study includes a large variety of single garments that can be combined to whole-body ensembles as needed. However, local effects of overlaying clothing items were not considered in this approach. Havenith et al. (2012) presented empirical equations based on the seasonal dressing customs of Europeans according to the outdoor air temperature to determine the local clothing insulation for seven body parts. This approach gives a rough estimation on the clothing insulation worn in a specific season of the year but does not include the properties of the worn garments, such as the clothing material or fit, and neglects the parameters of the indoor environment.

In conclusion, current literature does not provide enough clothing insulation values for a variety of typical office clothing ensembles under different air speeds and with body movement. To close this gap, we measured the local dry thermal resistance of eight body parts at three different air speeds and including body movement of a large variety of typical office clothing ensembles using a sweating agile manikin and a sweating foot manikin at Empa, Switzerland. Additionally, the local clothing area factors were estimated based on 3D scans of the clothing items. This paper presents the results of the measurements and discusses the effect of air speed, body movement and garment fit on the local thermal properties of the clothing ensembles.

## Methods

### Measuring equipment

The local dry thermal resistance (*I*_*T*,*i*_) of the office clothing ensembles was measured using the sweating agile manikin (SAM) (Richards and Mattle 2001) at Empa, Switzerland. The manikin consists of 22 shell elements, which are made from thin-walled aluminum-polyethylene composite. Moreover, nine guards (hands, feet, elbows, knees, and the face block) are installed to minimize the heat exchange between elements and the environment. All elements are uniformly and separately heated. The mean temperature of each shell part is measured at its outer surface with evenly distributed nickel resistance wires. Furthermore, SAM can be connected with a movement simulator, which enables the manikin to perform realistic movements of up to 2.5 km/h walking speed.

Because SAM has no foot segments, the *I*_*T*,*i*_ of representative shoe and sock combinations were measured with the foot simulator as described by Babic et al. (2008) and Bogerd et al. (2012). The foot manikin represents a right foot of size EU 43 and consists of 13 separately heated metal elements. Furthermore, walking can be simulated with a net force of up to 25 kg and with a maximum of 25 steps per minute.

Both manikins are placed in separate climate chambers. The chambers’ temperature and relative humidity can be controlled within ±1°C and ±5%, respectively.

### Garments and ensembles

**Fig. 1:**
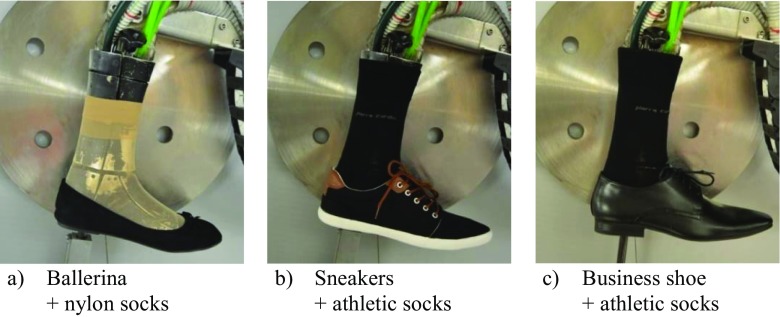
Shoe/sock combinations. **a** Ballerina + nylon socks. **b** Sneakers + athletic socks. **c** Business shoe + athletic socks

In this study, garments and their combinations typically worn in an office environment are considered. Since SAM has an average male statue, mainly male clothing items were chosen. The detailed properties of all clothing items are summarized Table S1 (supplementary information). To account for different preferences in clothing fit, the t-shirt, long-sleeved shirt (abbreviation: shirt), long-sleeved smart shirt (abbreviation: smart shirt), and jeans were included in different sizes, representing tight (T), regular (R), and loose (L) fits. The clothing items were combined to 23 whole-body and three foot combinations, which are summarized in Table 1 and Fig. 1.

**Table 1:**
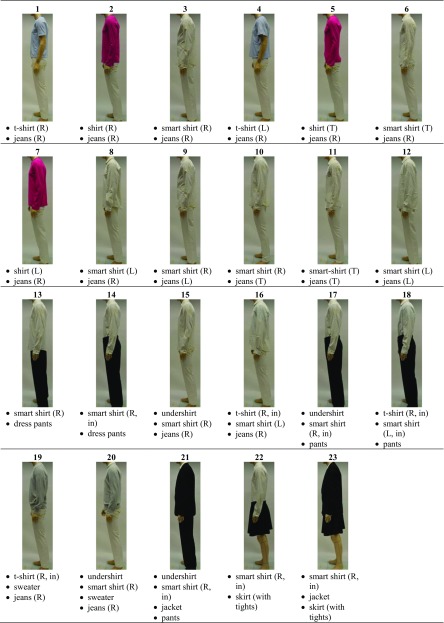
Whole-body clothing ensembles (all outfits include briefs)

*T* tight fit, *R* regular fit, *L* loose fit, *in* tucked in pants

### Local clothing area factors

The total clothing area factor *f*_*cl*_ accounts for the increase of the total body surface area by the addition of clothing and is defined as follows:

1 $$ {f}_{cl}=\frac{A_{\mathrm{dressed}}}{A_{\mathrm{nude}}} $$

where *A*_dressed_ is the outer surface area of the dressed body and *A*_nude_ is the surface area of a nude body (ISO 9920 2009). For the local clothing area factor *f*_*cl*,*i*_, this definition is applied to all body parts separately.

**Fig. 2:**
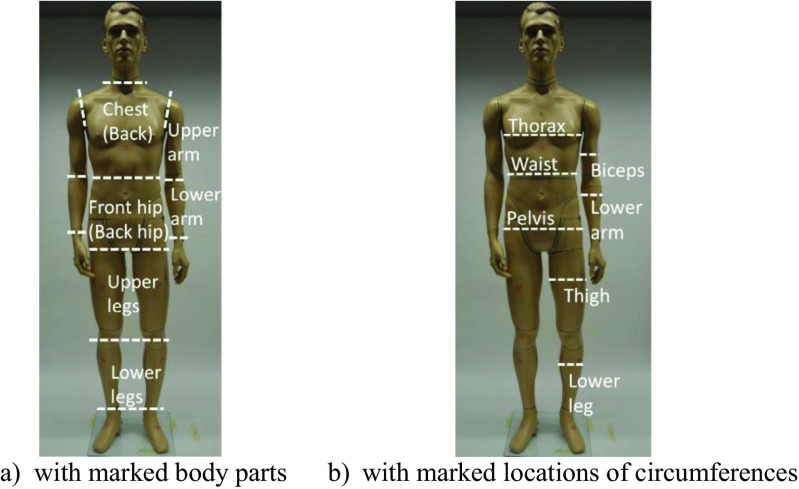
Manikin James used to obtain local clothing area factors. **a** With marked locations of circumferences. **b** With marked body parts

For obtaining *f*_*cl*,*i*_ of all used garments, a 3D scanner was used to scan the nude and dressed shop window manikin James to obtain the respective surface areas (Psikuta et al. 2012, 2015). In a 3D surface inspection software (Geomagic Control 2014, 3D Systems®, USA), the scans of the nude and dressed manikin were cut according to the defined body parts (Fig. 2a) before *f*_*cl*,*i*_ of the single sections were calculated. In all cases, uncovered areas, e.g., opening of the jacket on the chest, were not considered.

**Fig. 3:**
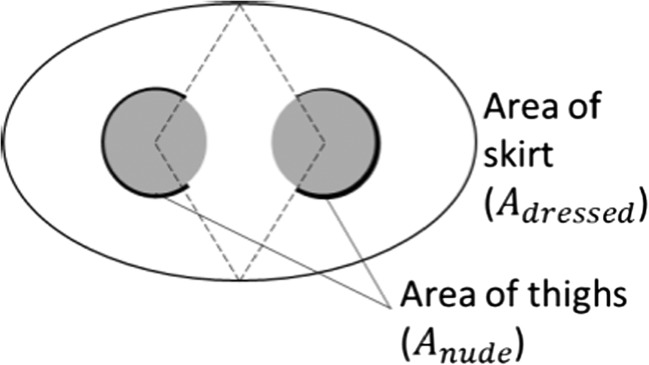
Schematic for obtaining the thigh area factor for a person wearing a skirt

A special case is *f*_*cl*,*i*_ of the skirt since the inner thighs are not covered by a fabric. In this paper, we decided to reduce the nude area of the thighs for the upright, stationary posture of the manikin, since the thighs are close to each other, and therefore, the heat loss is reduced in this area. The nude area of the thighs, in the described case, was determined by drawing a line from the middle front and back of the skirt to the center of the thigh (Fig. 3). Only the skin surface at the outer sides of the thighs (bold lines in Fig. 3) are used for calculating the nude skin area *A*_nude_ which is needed for computing *f*_*cl*,*i*_ (Eq. (1)). Hence, the inner parts of the thighs were left out. For the measurement with the moving manikin, the whole nude area of the thighs was considered.

For most garments, the surface areas were obtained for three scans. Between the scans, the manikin was redressed to account for differences in draping. All results of *f*_*cl*,*i*_ measured on James can be found in Table S2 and S3 of the supplementary information.

When considered strictly, *f*_*cl*,*i*_ depends on the garment fit at a specific body part. Hence, *f*_*cl*,*i*_ should be adjusted, when using other manikins, human subjects or garment fit. A measure of clothing fit is the ease allowance *EA* which is the difference between the circumferences of a clothing item (*CF*_cloth, *i*_) and the manikin (or person) (*CF*_man, *i*_) at a specific body landmark (Fig. 2b) (ISO 8559 1989).

2 $$ E{A}_i=C{F}_{\mathrm{cloth},i}-C{F}_{\mathrm{man},i} $$

The garments were marked and measured at the same positions. All EA were then calculated for all items at the relevant positions. For the skirt, only the EA of the hip was measured. The EA of the clothing items on James are summarized in Table 2. Negative values indicate that the clothing is locally stretched while wearing it. Since the geometry of other manikins and real persons is slightly different, a correction is needed. The *f*_*cl*,*i*_ for thermal manikin SAM are adjusted by measuring the respective circumferences and correcting the EA accordingly (see Table 3).

**Table 2:** Ease allowances of clothing items—James manikin

	Ease allowance (cm)														
	T-shirt—regular	T-shirt—loose	Shirt—tight	Shirt—regular	Shirt—loose	Smart shirt—tight	Smart shirt—regular	Smart shirt—loose	Sweater	Business jacket	Jeans—tight	Jeans—regular	Jeans—loose	Dress pants	Skirt
Biceps	2	10	1	3	9	9	11	14	6	12.5	–	–	–	–	–
Lower arm	–	–	− 1	0.5	3	7.5	8.5	11	7	11	–	–	–	–	–
Thorax	7	12	− 8	0	8	1	10	18	13	14	–	–	–	–	–
Waist	22	39	10	20	28	12	24	39	42	34	–	–	–	–	–
Pelvis	8	19	− 9	1	9	1	11	21	22/− 10*	23	6	6	9	14	20
Thigh	–	–	–	–	–	–	–	–	–	–	3	6	10	12	–
Lower leg	–	–	–	–	–	–	–	–	–	–	3.5	7.5	12.5	15.5	–

*EA of sweater at pelvis includes loosely falling main body of sweater (EA = 22 cm) and tight ribbed band (EA = − 10 cm)

**Table 3:** Circumferences of manikin James and SAM as well as correction for ease allowances

Location	Circumference on James (cm)	Circumference on SAM (cm)	Correction of ease allowance (cm)
Biceps	30	31	− 1
Lower arm	27.5	24	3.5
Thorax	101	102	− 1
Waist	74	78	− 4
Pelvis	94	93	1
Upper leg	53	58	− 5
Lower leg	35.5	39.5	− 4

The *f*_*cl*,*i*_ of the shoe/sock combinations were estimated using the more classical method of calculating the nude and dressed areas using photographs and post-processing them in suitable software, e.g., Photoshop Elements (Adobe Systems Software, Ireland) (McCullough et al. 1985; Havenith et al. 2015).

### Local dry thermal resistance

The local dry thermal insulation (*I*_*T*,*i*_) was measured according to standard EN-ISO 15831 (2004). For the measurements of the whole-body clothing ensembles on SAM, the surface temperature *T*_skin_ was set to 34°C, the operative temperature of the environment *T*_*op*_ to 21°C and the relative humidity to 40%. The environmental conditions were monitored using a measurement tree with the sensors placed in front of the manikin (ThermCondSys5500 and AirDistSys 5000, Sensor electronic, Poland). The operative temperature and relative humidity was obtained at the height of the waist and their standard deviation was typically around 0.1°C and 1%, respectively. The air speed was measured at three heights, namely ankles, waist, and head. The standard deviation for the air speed varied from about 0.02m/s for lowest air speed to 0.1m/s for largest air speed. In the reference case (test case 1), the air speed was set to 0.2m/s and SAM was in standing position. In these conditions, *I*_*T*,*i*_ of all clothing ensembles (Table 1) were measured. To analyze the influence of varying air speed and the addition of body movement, *I*_*T*,*i*_ of six outfits (nos. 1, 3, 14, 19, 21, 22) was determined in four additional test cases (TC 2–5). In test cases 2 and 3, SAM was in standing position and the air speed was set to 0.4m/s and to 1.0m/s, respectively. For the fourth and fifth test cases, SAM was connected to the moving simulator and air speeds of 0.2m/s and 1.0m/s were used, respectively. The walking speed of the movement simulator was controlled to 2.5 km/h . In all cases, the air was directed from the front of the manikin. The local thermal resistance of the air layer *I*_*a*, *i*_ was defined as the thermal insulation of the nude manikin. For each condition, three independent measurements were done for *I*_*a*, *i*_.

Each clothing ensemble was measured in the relevant test cases at least twice for 45 min. If the difference between the two measurements exceeded 4% for the total dry thermal resistance or 10% for the local dry thermal resistances, an additional repetition was conducted (ISO 15831 2004). In between the experiments, the manikin was redressed to account for differences in the draping of the garments. During the experiments, the dry heat loss ${\dot{Q}}_{loss,i}$, the skin temperature *T*_*skin*,*i*_ of all body parts *i*, and the environmental parameters were recorded. Then, *I*_*T*,*i*_ and the local intrinsic clothing insulation *I*_*cl*,*i*_ of a specific body part *i* were computed as an average of the last 20 min (steady state) using the measured *f*_*cl*,*i*_ as described in the sections “Local clothing area factors” and “Local clothing area factors” and Eqs. (3) and (4), respectively.

3 $$ {I}_{T,i}=\frac{T_{skin,i}-{T}_{op}}{{\dot{Q}}_{loss,i}}\kern0.75em \left({\mathrm{m}}^2\ \mathrm{K}/\mathrm{W}\right) $$

4 $$ {I}_{cl,i}={I}_{T,i}-\frac{I_{a,i}}{f_{cl,i}}\kern0.75em \left({\mathrm{m}}^2\ \mathrm{K}/\mathrm{W}\right) $$

In the case of the foot manikin, the skin temperature set point was set to 35 °C. The air speed in this climate chamber could not be altered. Measurements during the experiments showed that the air speed varied between 0.15m/s and 0.2m/s. The standard deviation of the operative temperature and relative humidity during the experiments in this chamber were 0.1°C and 1%, respectively. To investigate the effect of movement, measurements were performed on the static foot and on the moving foot with a speed of 25 steps per minute (about 1.2 km/h) and a pressure of 25 kg. A larger number of steps per minute would be closer to the walking speed of SAM but is not supported by the current system (Babic et al. 2008; Bogerd et al. 2012). Each shoe/ sock combination was then measured three times for 60 min in each scenario with changing shoes in between measurements. The resulting total clothing insulation *I*_*T*, foot_ was determined for the sectors of the foot manikin, which represent the actual foot (below ankle) and are mostly covered by the shoes and socks.

## Results and discussion

### Local clothing area factors

**Fig. 4:**
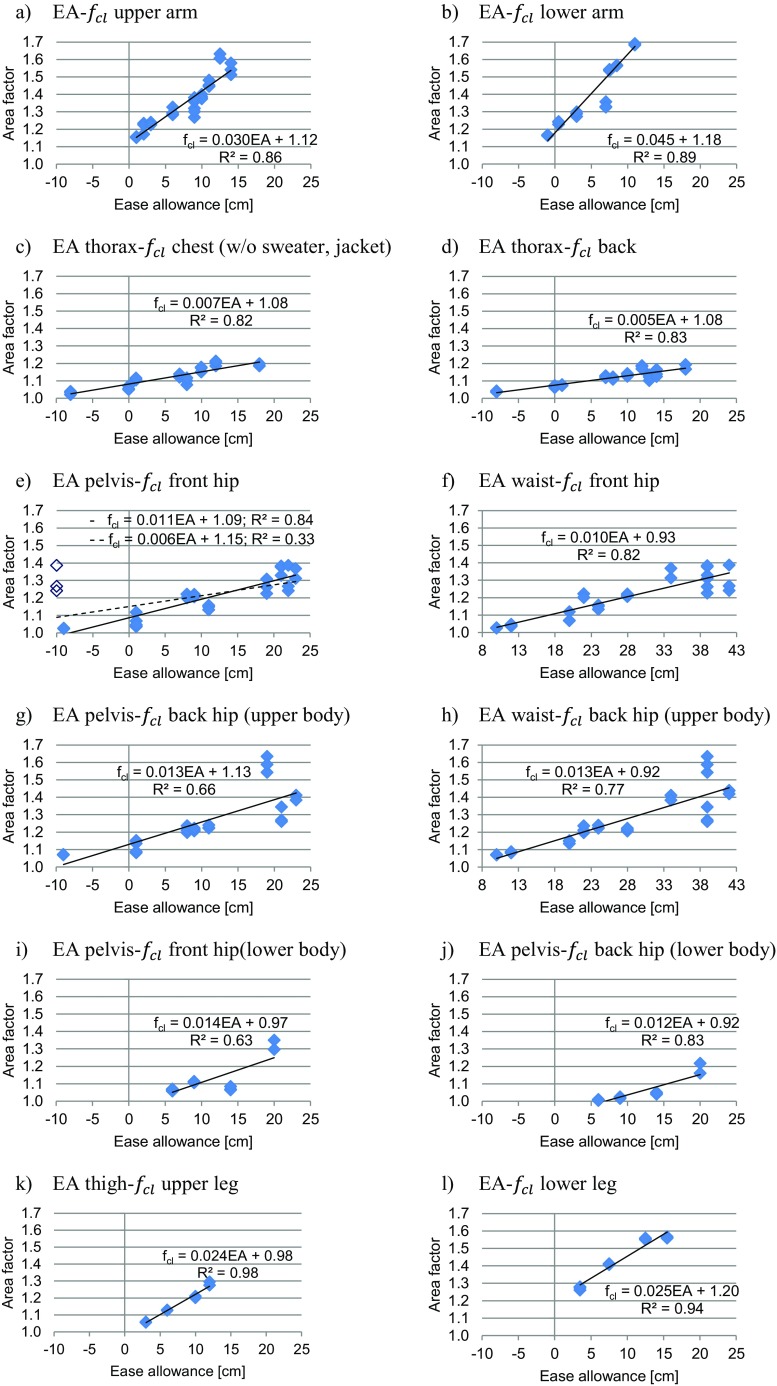
Correlation of local clothing area factors *f*_*cl*,*i*_ and ease allowances *EA*. **a** EA-*f*_*cl*_ upper arm. **b** EA-*f*_*cl*_ lower arm. **c** EA thorax-*f*_*cl*_ chest (w/o sweater, jacket). **d** EA thorax-*f*_*cl*_ back. **e** EA pelvis-*f*_*cl*_ front hip. **f** EA waist-*f*_*cl*_ front hip. **g** EA pelvis-*f*_*cl*_ back hip (upper body). **h** EA waist-*f*_*cl*_ back hip (upper body). **i** EA pelvis-*f*_*cl*_ front hip (lower body). **j** EA pelvis-*f*_*cl*_ back hip (lower body). **k** EA thigh-*f*_*cl*_ upper leg. **l** EA-*f*_*cl*_ lower leg

To obtain *f*_*cl*,*i*_ for the used garments on manikin SAM, the correlation between *f*_*cl*,*i*_ and the EA were investigated. In Fig. 4, the linear fitting and *R*^2^ values are shown for all body parts. High linear correlations (*R*^2^ value > 0.8) between *f*_*cl*,*i*_ and EA are seen for the upper and lower arm, back, back hip for lower body items as well as the lower and upper leg. For the chest, the *R*^2^ value is higher, when the sweater and jacket are excluded from the graph. This observation might be due to the design differences between the sweater, the jacket and the other shirts. For the EA pelvis - *f*_*cl*,*i*_ front hip correlation in Fig. 4e, two linear trend lines are shown, because the EA of the sweater includes the loosely falling main body of the sweater (EA = 22 cm, solid line) and the tight ribbed band (EA =  − 10 cm, dashed line). The squared Pearson coefficient is low for the linear correlation including the negative EA of the sweater (*R*^2^ = 0.33)and higher when the larger EA is taken into account (*R*^2^ = 0.84). Another option to predict *f*_*cl*,*i*_ of the front and back hip of the upper body is to use the correlation to the EA of the waist (Fig. 4f, h). Furthermore, the slope of the linear correlations varies for the different body parts. At the chest, for example, *f*_*cl*,*i*_ for different EA varies only from 1.05 to 1.20. In contrast, *f*_*cl*,*i*_ at the lower arm covers a range from 1.15 to 1.7. Hence, the importance to adjust *f*_*cl*,*i*_ with regard to EA depends on the body part. It can also be noted that *f*_*cl*,*i*_ of some body parts at the same body landmark is very similar. For example, this observation can be seen for *f*_*cl*,*i*_ of the chest and back at the thorax and for the front hip and back at the waist.

To estimate *f*_*cl*,*i*_ of the whole-body ensembles in relation to SAM (Table 4), the following procedure was applied:

- The linear correlations, as shown in Fig. 4a–d, f, h–l were applied.
- It is assumed that the outermost garment defines *f*_*cl*,*i*_ of a specific body part.
- In the case of SAM, the hip is defined from the waist downwards (Fig. 2), and hence, it is mainly covered by the upper body garment. Therefore, *f*_*cl*,*i*_ for the hip is taken mostly from the upper body garment. For the cases where the long-sleeved smart shirt is worn inside the dress pants, *f*_*cl*,*i*_ of the hips of these trousers is taken.
- Skirt: No equation can be used to calculate the reduction of *f*_*cl*,*i*_ because of the larger upper legs on SAM. For the jeans and pants, *f*_*cl*,*i*_ of the upper legs was reduced by 0.10 to 0.15. Therefore, *f*_*cl*,*i*_ of the upper leg of the skirt is also reduced from 2.29 to 2.15 for the stationary and from 1.5 to 1.35 for the moving manikin as an estimation.

**Table 4:** Estimated local clothing area factors (*f*_*cl,i*_) for different clothing ensembles for SAM

Outfit	Local clothing area factor							
	Upper arm	Lower arm	Chest	Back	Front hip	Back hip	Upper leg	Lower leg
1	1.15	–	1.13	1.11	1.11	1.15	1.01	1.29
2	1.18	1.36	1.07	1.07	1.09	1.13	1.01	1.29
3	1.42	1.72	1.16	1.12	1.13	1.18	1.01	1.29
4	1.39	–	1.17	1.13	1.28	1.37	1.01	1.29
5	1.12	1.29	1.01	1.03	1.01	1.01	1.01	1.29
6	1.36	1.68	1.08	1.08	1.01	1.02	1.01	1.29
7	1.37	1.47	1.14	1.11	1.17	1.23	1.01	1.29
8	1.51	1.83	1.22	1.17	1.28	1.37	1.01	1.29
9	1.42	1.72	1.16	1.12	1.13	1.18	1.10	1.42
10	1.42	1.72	1.16	1.12	1.13	1.18	1.01	1.19
11	1.37	1.67	1.09	1.08	1.00	1.03	1.01	1.19
12	1.51	1.83	1.22	1.17	1.28	1.37	1.10	1.42
13	1.42	1.72	1.16	1.12	1.13	1.18	1.15	1.49
14	1.42	1.72	1.16	1.12	1.18	1.09	1.15	1.49
15	1.42	1.72	1.16	1.12	1.13	1.18	1.01	1.29
16	1.51	1.83	1.22	1.17	1.28	1.37	1.01	1.29
17	1.42	1.72	1.16	1.12	1.18	1.09	1.15	1.49
18	1.51	1.83	1.22	1.17	1.18	1.09	1.15	1.49
19	1.27	1.65	1.18	1.14	1.31	1.41	1.01	1.29
20	1.27	1.65	1.18	1.14	1.31	1.41	1.01	1.29
21	1.46	1.83	1.19	1.15	1.23	1.30	1.15	1.49
22	1.42	1.72	1.16	1.12	1.13	1.18	2.15/1.35*	1.00
23	1.46	1.83	1.19	1.15	1.23	1.30	2.15	1.00

*Local clothing area factor of the skirt for stationary measurements (2.15) and in case of movement (1.35)

For the shoe/sock combinations, *f*_*cl*,*i*_ were approximated with the 2D photo method and are as follows:

- Ballerina/ nylon socks: 1.2
- Sneakers/ athletic socks: 1.4
- Business shoes/ athletic socks: 1.3

For tight clothing items, the described method can lead to calculated *f*_*cl*,*i*_ below 1. This situation happens when the circumference of a specific body part on a manikin or human subject is larger than the one of the original manikin (James), so that the EA for the larger body becomes negative. For example, the calculated *f*_*cl*,*i*_ of the tight jeans on the upper leg (outfit 10 and 11) and the tight long-sleeved shirt on the front hip (outfit 5) were 0.94 and 0.99, respectively. However, in reality the garment will stretch and the minimum value can only be the circumference of the specific body part with added thickness of the fabric. For the tight jeans on the upper leg the calculation reads *f*_*cl*, *i*, *min*_ = (58 cm + 2 ∙ π · 0.067 cm)/58 cm = 1.007 and for the tight long-sleeved shirt on the hip it is *f*_*cl*, *i*, *min*_ = (93 cm + 2 ∙ π · 0.087 cm)/93 cm = 1.006. Hence, these minimal values were used for further calculations.

#### Comparison to values found in the literature

As mentioned in the “Introduction,” there are very few values for *f*_*cl*,*i*_ in the literature. In fact, the ones found in Nelson et al. (2005) are attributed to entire clothing items, and not to single body parts. Since the air gaps between the clothing item and the body surface can vary for different body parts, this may lead to false values for some body parts covered by the clothing item, especially if its area is small compared to the area of the whole garment. For example, *f*_*cl*,*i*_ of the long-sleeved shirt with shirt collar in Nelson et al. (2005) is 1.24. This value is slightly higher but comparable to *f*_*cl*,*i*_ of the chest, back, front and back hip of the regular long-sleeved smart shirt in this paper (1.12–1.17). However, it is much lower than *f*_*cl*,*i*_ of the upper and lower arm (1.43 and 1.71, respectively). Another issue is that the fit of the clothing items are described very briefly with general terms such as “fitted” or “loose.” The straight, long trousers in Nelson et al. (2005), for instance, have a *f*_*cl*,*i*_ of 1.20 in the “fitted” case. However, this value is by 0.1 to 0.2 larger than the values obtained for the measured tight jeans (outfits 10–11) and also the regular fitting jeans (outfits 1–8) of this study on the hip and upper leg. A larger *f*_*cl*,*i*_ would mean that a smaller adjacent air insulation value is subtracted from the measured total thermal insulation of garment to calculate the intrinsic clothing insulation of a specific body part (see Eq. (4)). For example, the upper leg has an air layer insulation of 0.08 m^2^ K/W. *f*_*cl*,*i*_ of 1.0 and 1.2 would lead to the subtraction of 0.08 m^2^ K/W or 0.067 m^2^ K/W of air insulation, respectively, which is a difference of 16% in the intrinsic clothing insulation. Hence, a more exact measurement for the fit of clothing items, such as the ease allowance may help to avoid the mentioned inaccuracies.

### Dry thermal resistance

The results for *I*_*cl*,*i*_ of the whole-body clothing ensembles and *I*_*a*, *i*_ of all test cases are shown in Table 5. For the sock/shoe combinations, *I*_*T*,*i*_ in the non-moving case is 0.11 m^2^ K/W for the ballerinas with nylon socks and 0.13 m^2^ K/W for both the sneakers and business shoes combined with the athletic socks. These values are reduced by movement to 0.09 m^2^ K/W and 0.12 m^2^ K/W, respectively. The air layer insulation could only be determined for the non-moving foot and is 0.09 m^2^ K/W. Hence, *I*_*cl*,*i*_ of the sneakers, business shoes and ballerinas for the non-moving foot manikin can be computed by Eq. (4). Their values are 0.07 m^2^ K/W, 0.06 m^2^ K/W, and 0.04 m^2^ K/W, respectively.

**Table 5:** Results for local intrinsic clothing insulation of all outfits and local air layer insulation for all test cases

Outfit no./test case	Posture	Air-speed (m/s)	Upper arm	Lower arm	Chest	Back	Front hip	Back hip	Upper leg	Lower leg
			Local intrinsic clothing insulation (m^2^K/W)							
1	Standing	0.2	0.081	n/a	0.092	0.172	0.129	0.153	0.062	0.091
	Standing	0.4	0.079	n/a	0.090	0.158	0.137	0.182	0.054	0.090
	Standing	1	0.071	n/a	0.078	0.167	0.094	0.183	0.056	0.092
	Moving	0.2	0.061	n/a	0.084	0.212	0.125	0.135	0.043	0.068
	Moving	1	0.052	n/a	0.071	0.165	0.082	0.164	0.039	0.068
2	Standing	0.2	0.078	0.069	0.075	0.143	0.153	0.211	0.056	0.089
3	Standing	0.2	0.122	0.093	0.115	0.192	0.181	0.213	0.065	0.090
	Standing	0.4	0.116	0.088	0.105	0.183	0.151	0.198	0.060	0.089
	Standing	1	0.097	0.081	0.079	0.156	0.108	0.209	0.056	0.082
	Moving	0.2	0.089	0.076	0.084	0.234	0.132	0.171	0.047	0.070
	Moving	1	0.075	0.065	0.063	0.177	0.089	0.182	0.048	0.068
4	Standing	0.2	0.119	0.000	0.103	0.178	0.162	0.192	0.055	0.088
5	Standing	0.2	0.066	0.062	0.051	0.110	0.122	0.156	0.054	0.087
6	Standing	0.2	0.102	0.095	0.072	0.161	0.120	0.157	0.056	0.089
7	Standing	0.2	0.112	0.076	0.095	0.164	0.168	0.213	0.062	0.089
8	Standing	0.2	0.148	0.095	0.122	0.215	0.186	0.227	0.069	0.090
9	Standing	0.2	0.125	0.091	0.117	0.189	0.191	0.213	0.102	0.092
10	Standing	0.2	0.124	0.091	0.114	0.196	0.149	0.196	0.052	0.067
11	Standing	0.2	0.104	0.091	0.077	0.159	0.107	0.155	0.043	0.067
12	Standing	0.2	0.150	0.097	0.128	0.228	0.214	0.228	0.100	0.093
13	Standing	0.2	0.131	0.090	0.120	0.212	0.181	0.198	0.156	0.080
14	Standing	0.2	0.129	0.092	0.120	0.202	0.140	0.142	0.153	0.082
	Standing	0.4	0.125	0.093	0.105	0.179	0.135	0.135	0.142	0.081
	Standing	1	0.106	0.088	0.085	0.159	0.108	0.143	0.156	0.087
	Moving	0.2	0.079	0.069	0.087	0.212	0.109	0.140	0.072	0.048
	Moving	1	0.074	0.066	0.066	0.176	0.079	0.108	0.059	0.045
15	Standing	0.2	0.129	0.095	0.147	0.268	0.192	0.241	0.071	0.088
16	Standing	0.2	0.184	0.094	0.180	0.316	0.224	0.277	0.074	0.092
17	Standing	0.2	0.120	0.084	0.144	0.296	0.174	0.209	0.166	0.087
18	Standing	0.2	0.194	0.098	0.177	0.324	0.188	0.196	0.160	0.086
19	Standing	0.2	0.183	0.099	0.157	0.303	0.246	0.274	0.062	0.087
	Standing	0.4	0.164	0.095	0.152	0.311	0.182	0.217	0.061	0.093
	Standing	1	0.159	0.089	0.139	0.309	0.145	0.259	0.055	0.089
	Moving	0.2	0.140	0.083	0.148	0.393	0.226	0.290	0.054	0.069
	Moving	1	0.124	0.064	0.119	0.282	0.149	0.217	0.048	0.070
20	Standing	0.2	0.158	0.122	0.187	0.379	0.232	0.265	0.065	0.089
21	Standing	0.2	0.287	0.172	0.283	0.505	0.330	0.368	0.206	0.085
	Standing	0.4	0.284	0.164	0.279	0.574	0.299	0.416	0.206	0.088
	Standing	1	0.243	0.151	0.205	0.520	0.166	0.347	0.183	0.084
	Moving	0.2	0.175	0.094	0.216	0.587	0.213	0.273	0.071	0.046
	Moving	1	0.161	0.095	0.177	0.535	0.158	0.299	0.074	0.045
22	Standing	0.2	0.122	0.094	0.120	0.183	0.143	0.144	0.175	0.010
	Standing	0.4	0.122	0.095	0.108	0.178	0.137	0.142	0.150	0.007
	Standing	1	0.098	0.075	0.081	0.159	0.110	0.136	0.127	0.005
	Moving	0.2	0.079	0.073	0.083	0.188	0.142	0.157	0.071	0.006
	Moving	1	0.076	0.067	0.068	0.177	0.111	0.155	0.074	0.007
23	Standing	0.2	0.298	0.166	0.270	0.515	0.296	0.354	0.207	0.006
			Local air layer insulation (m^2^K/W)							
TC1	Standing	0.2	0.09	0.06	0.10	0.15	0.06	0.07	0.08	0.07
TC2	Standing	0.4	0.07	0.05	0.08	0.15	0.05	0.07	0.07	0.06
TC3	Standing	1	0.05	0.03	0.05	0.10	0.03	0.05	0.04	0.04
TC4	Moving	0.2	0.09	0.05	0.10	0.18	0.06	0.07	0.07	0.05
TC5	Moving	1	0.05	0.03	0.05	0.11	0.04	0.05	0.04	0.04

#### Comparison to values found in the literature

For a clothing ensemble consisting of a t-shirt and jeans, the measured *I*_*cl*,*i*_ of this study, namely outfit 1 and 4 (with regular and loose t-shirt, respectively), can be compared to four other studies (Nelson et al. 2005; Havenith et al. 2012; Lee et al. 2013; Lu et al. 2015) as shown in Table 6. For the empirical equations by Havenith et al. (2012) an air temperature of 22°C is assumed. The comparison in Table 6 reveals that the differences in *I*_*cl*,*i*_ vary depending on the body part. For example, *I*_*cl*,*i*_ of the upper arm found in the literature are comparable to ones measured in this study. For the back, the values from the literature are generally smaller than from outfit 1 or 4. The most variance between the studies and our values is seen at the front and back hip. These observed variations might be caused by the distinct material and fit of the garments. Garments made of thicker material and with a looser fit, i.e., larger air gap, would result in increased clothing insulation values. Moreover, the differences in the construction, setup, and posture of the used manikins can affect the result. For example, SAM has pronounced anatomical shoulder blades, unlike most of the other thermal manikins with simplified or smoothed body shapes, which creates a higher clothing insulation through a larger air gap when the clothing is on. Moreover, the manikin in Lee et al. (2013) was in sitting position, which causes smaller air gaps at the back, pelvis, thigh, and calf. Also, in a sitting position, the draping of the clothing is different than in upright position (Mert et al. 2017). Another issue comparing different studies is that the body parts are defined differently. This difference especially occurs for the torso. In Lee et al. (2013), for instance, the torso is divided in the chest, back and pelvis, whereas in Lu et al. (2015) it consists of the chest, back, abdomen and pelvis. In these regions, the clothing draping pattern can differ as discussed by Frackiewicz-Kaczmarek et al. (2015). Another factor that can cause different *I*_*cl*,*i*_ is slight variations in the environmental conditions. For example, the air speed in the studies by Lee et al. (2013) and Lu et al. (2015) is 0.1m/s and 0.15m/s, respectively, whereas the air speed in this study was set to 0.2m/s. However, the compared papers do not contain all of this information. Hence, it is difficult for users of thermophysiological or thermal sensation models to extract the most suitable set of *I*_*cl*,*i*_ for a specific simulation case.

**Table 6:** Comparison of local intrinsic clothing thermal resistance for a light clothing ensemble (Nelson et al. 2005; Havenith et al. 2012; Lee et al. 2013; Lu et al. 2015)

	Local clothing insulation (m^2^K/W)					
Body part	Lee (No. 8)	Lu (EN 9)	Nelson and Curlee	Havenith (22 °C)	Measured outfit 1	Measured outfit 4
Chest	0.18	0.17	0.10	0.12	0.09	0.10
Back	0.13	0.12	0.10	0.12	0.17	0.18
Upper arm	0.07	0.07	0.10	0.12	0.08	0.12
Front hip	0.16	0.17	0.24	0.12	0.13	0.16
Back hip		0.22	0.24	0.12	0.15	0.19
Thigh	0.09	0.09	0.08	0.13	0.06	0.05
Lower leg	0.10	0.08	0.13	0.13	0.09	0.08
					Business shoes/sneakers	
Feet	0.13	–	0.22	0.08	0.06/0.07	

The intrinsic clothing insulation of the business shoes and sneakers are also included in Table 6 and compared to the values found in the mentioned studies. In general, the variation of the values for the foot dry insulation is high with this study’s values being the lowest. The range of the measured values (excluding the value by Nelson et al. (2005)) is 0.6–0.13 m^2^K/W. In an inter-laboratory test on thermal foot manikins by Kuklane et al. (2005), the effective insulation values also varied by ± 0.3–0.6 m^2^K/W depending on the tested shoe/sock combination. When compared to the measurements on army boots on the same foot manikin by Bogerd et al. (2012), *I*_*T*,*i*_ of the business shoes and sneakers are in line with *I*_*T*,*i*_ of army boots which was around 0.18 m^2^K/W.

For the ballerinas, no comparable values were found. In Lee et al. (2013), the sandals have a local intrinsic insulation value of around 0.4 *clo* (0.06 m^2^K/W), and in Kuklane et al. (2009), the described sandal has an effective insulation (*I*_*T*,*i*_ − *I*_*a*, *i*_) of 0.06 m^2^K/W. In both cases, the values are higher than for the ballerinas, even though a similar range would be expected. Three issues can be raised on these shoe insulation measurements. Firstly, the two sandals touched the ground during measurements, whereas the non-moving foot was hanging free. Hence, there was no convection on the sole of the sandals, which can result in overall higher *I*_*T*,*i*_. Secondly, the sandals of the study by Kuklane et al. (2009) have the highest effective sole insulation (0.261 m^2^K/W) in the study, which also results in relatively high *I*_*T*,*i*_ for the whole sandals. Lastly, the shoe insulation depends on the included sectors of the foot manikin in the calculation. For this study, the sectors of the foot manikin below the ankle were used. However, the ballerinas, for instance, do not cover the dorsal foot, which means that *I*_*T*,*i*_ of the whole foot will be less than *I*_*T*,*i*_ of the covered areas. In our case, the values are 0.11 m^2^K/W and 0.13 m^2^K/W, respectively. Conclusively, these issues should be considered and reported in studies on shoe insulation measurements to be able to do a fair comparison. This information will also help to choose the best values to use in thermophysiological models.

#### Effect of air speed and body movement

**Fig. 5:**
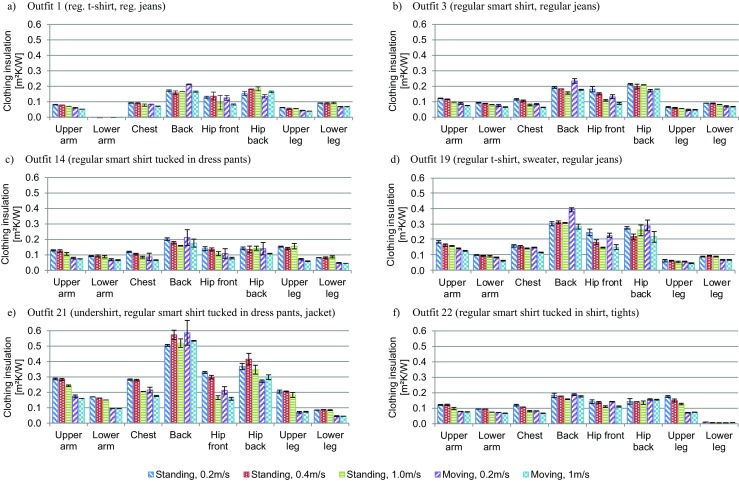
Influence of air speed and body movement on local intrinsic clothing insulation. **a** Outfit 1 (reg. t-shirt, reg. jeans). **b** Outfit 3 (regular smart shirt, regular jeans). **c** Outfit 14 (regular smart shirt tucked in dress pants). **d** Outfit 19 (regular t-shirt, sweater, regular jeans). **e** Outfit 21 (undershirt, regular smart shirt tucked in dress pants, jacket). **f** Outfit 22 (regular smart shirt tucked in shirt, tights)

For six outfits (1, 3, 14, 19, 21, 22), *I*_*cl*,*i*_ was obtained for two additional air speeds and body movement (Table 5, Fig. 5). Also, the correction factors were calculated using test case 1 as the reference and can be found in Table S4 to Table S7 in the supplementary materials. Additionally, the graphs showing the influence of increased air speed and the addition of body movement on *I*_*T*,*i*_ are given in Fig. S1. In general, the effect of increased air speed and body movement are more pronounced, but comparable, in *I*_*T*,*i*_, because the air layer is excluded for *I*_*cl*,*i*_. For the upper and lower arm, chest, front hip, upper and lower leg the increase in air speed and addition of body movement mostly reduced *I*_*cl*,*i*_. At an air speed of 0.4m/s, the reduction is minor and mostly within the standard deviation of *I*_*cl*,*i*_. For an air speed of 1.0m/s, low influence can be found for the upper and lower leg (except when a skirt is worn), whereas the reduction can reach up to 30–40% at the arms and chest. The effect of body movement is generally larger than for the increase in air speed for these body parts. This observation is more pronounced for upper legs with pants than with jeans. For the chest and front hip, the increase to an air speed of 1.0m/s or the addition of body movement yielded similar results. The differences of the effect of body movement on the reduction in *I*_*cl*,*i*_ might be caused by differences in the pumping effect, which is more pronounced for looser fitting clothing and on moving body parts. Hence, the pumping effect will be smaller for the relatively small movement of the torso when the manikin is in motion mode, and larger at the arms and legs, especially for looser fitting garments such as the pants. For the back and back hip, the results are more diverse. Surprisingly, the increase of air speed and body movement leads to an increase in *I*_*cl*,*i*_ in a large number of cases. This outcome might be caused by two possible issues: (1) as mentioned above, the anatomy of SAM results is an unnaturally hollow back and (2) the direction of the wind from the front to the back results in the displacement of the garments towards the back. In both cases, the air gap between the manikin’s skin and garment(s) will increase for higher air speeds until a critical speed is reached, where the air will leave through the lower opening of the shirt. Due to the hollow back, this critical wind speed value might be relatively high compared to other manikins, causing the rise in *I*_*cl*,*i*_ for at least a wind speed of 0.4m/s. The addition of body movement might emphasis this effect for low wind speeds, since the upper garment might to be push upwards creating a larger air gap due to the attachment of the legs to the moving simulator.

In the published literature for overall clothing insulation, general equations are given for the reduction of *I*_*T*_ due increased air speeds and body movement (Nilsson 1997; Nilsson et al. 2000; Havenith and Nilsson 2004; ISO 9920 2009). However, the effect of increased air speed and the addition of body movement cannot be generalized for our measurements, i.e., the local total and intrinsic clothing insulation. For the thermo-physiological modeling, this finding means that the effect of increased air speed and body movement should be considered separately for each body part rather than using a general reduction factor for the whole body as suggested in ISO 9920 (2009).

#### Effect of clothing fit

**Fig. 6:**
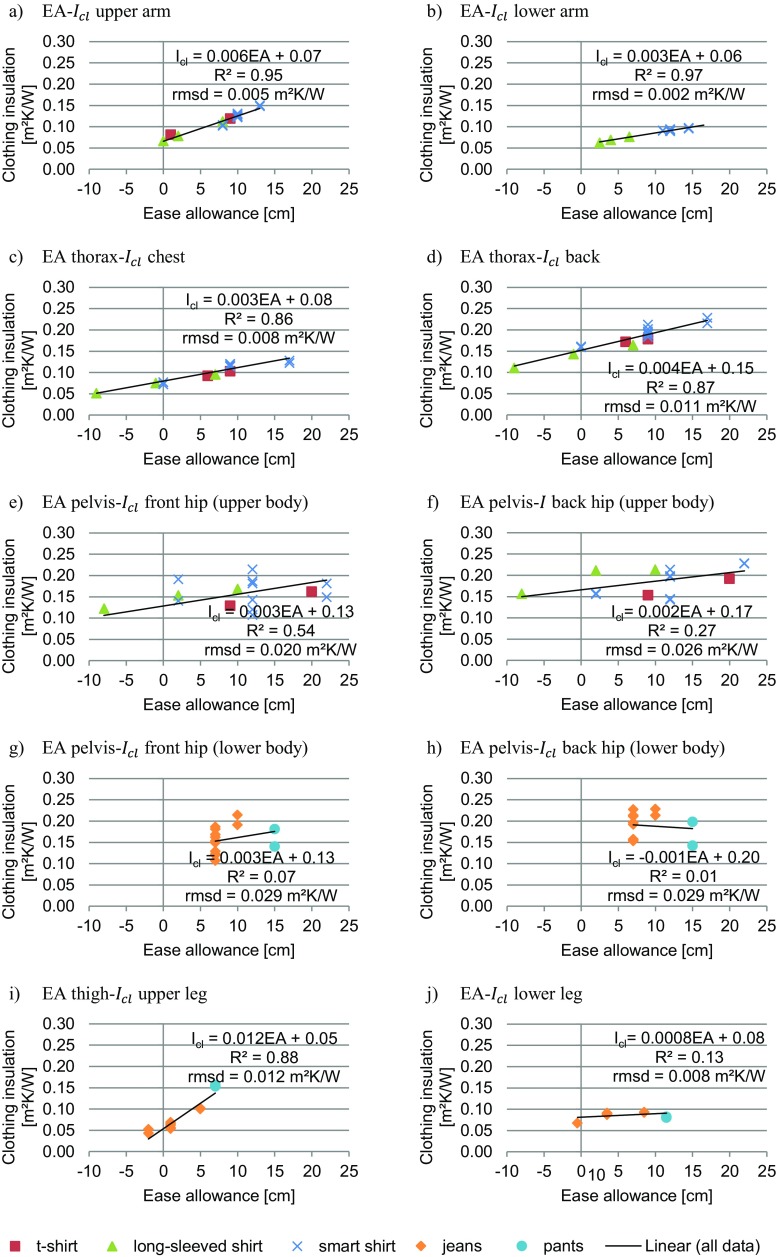
Correlation of local intrinsic clothing insulation and ease allowances. **a** EA-*I*_*cl*_ upper arm. **b** EA-*I*_*cl*_ lower arm. **c** EA thorax-*I*_*cl*_ chest. **d** EA thorax-*I*_*cl*_ back. **e** EA pelvis-*I*_*cl*_ front hip (upper body). **f** EA pelvis-*I* back hip (upper body). **g** EA pelvis-*I*_*cl*_ front hip (lower body). **h** EA pelvis-*I*_*cl*_ back hip (lower body). **i** EA thigh-*I*_*cl*_ upper leg. **j** EA-*I*_*cl*_ lower leg

For this study, the four clothing items t-shirt, long-sleeved shirt, long-sleeved smart shirt, and jeans were available in different fits. In Fig. 6, their *I*_*cl*,*i*_ are shown in relation to their EA at the respective body landmarks for outfits with one clothing layer. In most cases, *I*_*cl*,*i*_ increases for larger EA. These differences are relatively small for the lower arm, lower leg and back hip, but larger for the upper arm, chest, back, front hip, and upper leg. The *I*_*cl*,*i*_ of the smart shirt at the hips have more variation because it is combined with a larger variety of lower body garments, which overlap at the hips. Therefore, it is concluded that the knowledge of the exact fit, i.e. measurement of EA, is important when *I*_*cl*,*i*_ values are published.

#### Correlation of local ease allowance and local clothing insulation

In this study, *I*_*cl*,*i*_ of specific garments were measured. To apply these results to other research projects with similar outfits, the correlation between the local EA and *I*_*cl*,*i*_ values for single layer outfits was investigated. In our range of EA the trend might be approximated as linear, because EA is linearly correlated to air gap thickness (Frackiewicz-Kaczmarek et al. 2015; Mert et al. 2017) and air gap thickness, in turn, is close-to-linearly correlated to the heat transfer coefficient for the air gap range of 4 – 32 mm as investigated by Mert et al. (2017). The range of air gap thickness investigated in this paper is well covered by this study, and Fig. 6 provides the linear correlation, *R*^2^ values and root-mean-square deviation (rmsd) for all body parts.

High linear correlations (*R*^2^ > 0.85) and low rmsd values can be found for the upper and lower arm, chest, back, and upper leg. Hence, *I*_*cl*,*i*_ could be estimated using the provided linear correlations for other garments, manikins or human subjects. In contrast, the linear correlation at the front and back hip is weak (*R*^2^ < 0.6) and the results are more diverse. The main reason is that the clothing items of the upper and lower body overlap at this body part. Hence, a second air gap influences the result, which is not represented by EA measurement. The effect of this air gap can, for example, be seen in the difference of the regular smart shirt being tucked in the dress pants or not (0.14 vs 0.18 m^2^K/W, respectively; Table 5). However, the *R*^2^ value does not increase much if EA of the waist is used or only the upper body garments worn with the regular jeans are considered. Hence, for estimating *I*_*cl*,*i*_ at the hip for other garments, further factors, e.g., width of second air gap, should be considered. In the case of the lower leg, *I*_*cl*,*i*_ is very similar for all clothing items regardless of their fit (low *R*^2^ and low rsmd). Hence, *I*_*cl*,*i*_ for the lower leg cannot be predicted by a correlation equation, but might be assumed to be approximately 0.08 m^2^K/W. One reason for this result might be that the shape of the lower leg is very versatile ranging from 23 to 39 cm. The EA was measured at the widest place, and according to Table 2, there was a large selection of different EA. This is also shown by the steeper slope of *f*_*cl*,*i*_ vs EA in Fig. 4l. However, thermally, it seems that even for small EA the air gap at the lower part of the lower leg were relatively large. According to the dry heat transfer theory, the change in heat loss is minimal for further increase in air gap (Mert et al. 2016, 2017). Hence, the heat loss for all trousers is comparable.

The found correlations provide only an estimation of *I*_*cl*,*i*_ values for single layer outfits. For clothing ensembles with multiple layers, a correlation to EA cannot be expected, because this measurement does not include information about the number of layers and their air gaps. In future research, it could be investigated if an additional clothing layer would result in a similar increase in *I*_*cl*,*i*_ for a variety of single layer clothing ensembles.

#### Future research

In this paper, the local clothing insulation and local clothing area factors of a number of typical office clothing ensembles are calculated and analyzed for an upright position of the manikin SAM. However, a typical office situation also includes the sitting position. The measurements on SAM for this posture could not be conducted due to technical reasons. The largest influence of a sitting position can be expected for the contact areas with the chair, namely back hip and upper legs, and minor changes might be expected due to variations in draping of the clothing on the other body parts (Mert et al. 2016, 2017). Two effects are to be expected at these body parts: (1) the air layer between the skin and the clothing is reduced, and (2) the clothing insulation is influenced by the insulation of the chair. The first point was investigated by Mert et al. (2017). The second point raises the question regarding the kind of chair to be used and if measurements should be done for several chairs. Also, it might be inquired if the effect of the chair can be generalized.

This study also discusses the effect of the wind directed from the front to the back of the manikin on the resulting, relatively large, clothing insulation values of the back. Hence, future research may consider to vary the direction of the air to investigate the effect on the results. Then, the most appropriate value for a certain situation or the average might be considered depending on the application.

## Conclusion

This study extends the database of local clothing insulation and local clothing area factors of typical office clothing ensembles. For the local clothing area factors, empirical equations are provided to adjust the value for different garments, manikins or human subjects using the ease allowance as a reference. The local clothing insulation of most body parts are decreased by increased air speed and added body movement. However, the reduction is different for all body parts and therefore, cannot be generalized. Moreover, the fit of the garments also influences the local clothing insulation value. It is suggested to use the ease allowance for specifying the exact fit, rather than using general terms such as “fitted” or “loose.” In the case of single layer clothing combinations, the local clothing insulation correlates linearly to the ease allowance for most body parts covered with a single layer. In general, this study emphasizes the need for well documented measurements to get reproducible results and to choose accurate clothing parameters for thermo-physiological and thermal sensation modeling.

## Electronic supplementary material

ESM 1 (PDF 597 kb)
