# Supplementary material for: Local clothing thermal properties of typical office ensembles under realistic static and dynamic conditions
Source: Int J Biometeorol. 2018 Oct 29;62(12):2215–29. doi: 10.1007/s00484-018-1625-0 (PMC6244820; doi:10.1007/s00484-018-1625-0)
Supplement: Supplementary file 1 — (PDF 597 kb) [file 484_2018_1625_MOESM1_ESM.pdf]

Online supplementary information for the following article published in *International Journal of Biometeorology*

Local clothing thermal properties of typical office ensembles under realistic static and dynamic conditions

Stephanie Veselá[1], Agnes Psikuta[2], Arjan JH Frijns[1]

[1] Department of Mechanical Engineering, Eindhoven University of Technology, P.O. Box 513, 5600 MB Eindhoven, The Netherlands.

[2] Empa, Swiss Federal Laboratories for Materials Science and Technology, Lerchenfeldstr. 5, 9014 St. Gallen, Switzerland.

emails: s.vesela@tue.nl; agnes.psikuta@empa.ch; a.j.h.frijns@tue.nl

Table S1 Garments and their properties

Item	Briefs	Undershirt	T-shirt	Long-sleeved shirt	Long-sleeved smart shirt
Fits			Regular/ Loose	Tight/ Regular/ Loose	Tight/ Regular/ Loose
Picture	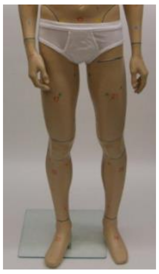	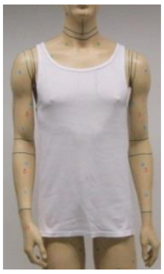	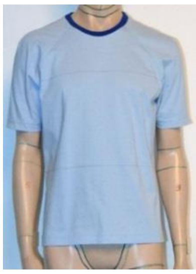	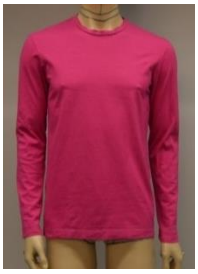	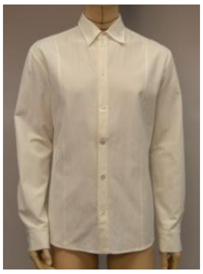
Fibre content	100% Cotton	100% Cotton	95% Cotton/ 5% Elastane	98% Cotton / 2% Spandex	100% Cotton
Mass per unit area	145.13 g/m³	145.13 g/m³	176 g/m³	188 g/m³	137 g/m³
Thickness	0.454 mm	0.454 mm	0.633 mm	0.87 mm	0.71 mm

Item	Sweater	Jacket	Jeans	Pants	Skirt
Fits			Tight/ Regular/ Loose		
Picture	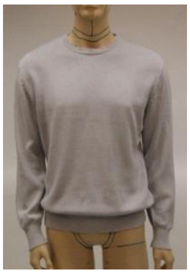	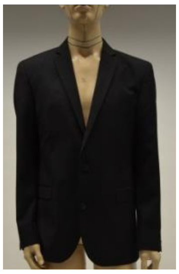	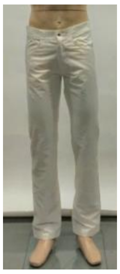	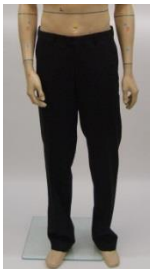	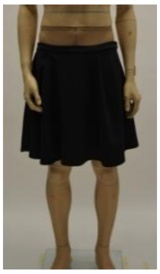
Fibre content	100% Cotton	65 % Polyester/ 35% Viskose	100% Cotton	60% Wool/ 38% Polyester/ 2% Elastane	93% Polyester/ 7% Elastane
Mass per unit area	361.58 g/m³	unknown	179 g/m³	202.15 g/m³	unknown
Thickness	1.680 mm	unknown	0.67 mm	0.416 mm	unknown

Item	Athletic sock	Nylon socks	Sneakers	Business shoes	Ballerinas
Picture	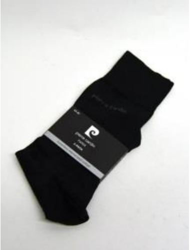	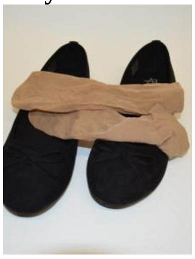	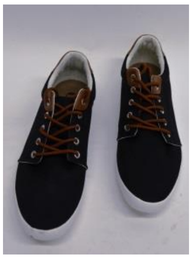	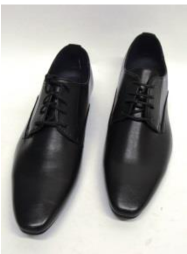	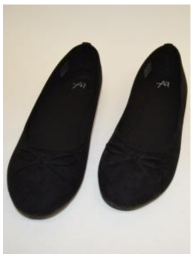
Fibre content	75% Cotton/ 25% Polyamid	100% Polyamid			
Mass per unit area					
Thickness					

Table S2 Local area factors mesured on manikin James – Part 1

Item	Body part	Local area factor James				
		1	2	3	avg	SD
Tshirt regular	Upper arm	1.23	1.17	1.23	**1.21**	0.03
	Chest	1.13	1.12	1.14	**1.13**	0.01
	Front hip	1.22	1.20	1.20	**1.21**	0.01
	Back	1.13	1.12	1.12	**1.12**	0.01
	Back hip	1.20	1.21	1.24	**1.21**	0.02
Tshirt loose	Upper arm	1.38	1.40	1.37	**1.39**	0.01
	Chest	1.18	1.21	1.21	**1.20**	0.01
	Front hip	1.23	1.31	1.26	**1.27**	0.03
	Back	1.19	1.18	1.17	**1.18**	0.01
	Back hip	1.63	1.54	1.59	**1.59**	0.04
Shirt tight	Upper arm	1.15	1.16	1.15	**1.15**	0.00
	Lower arm	1.17	1.16	1.16	**1.16**	0.00
	Chest	1.02	1.04	1.03	**1.03**	0.01
	Front hip	1.02	1.03	1.03	**1.03**	0.00
	Back	1.04	1.03	1.04	**1.04**	0.00
	Back hip	1.07	1.07	1.07	**1.07**	0.00
Shirt regular	Upper arm	1.22	1.24	1.23	**1.23**	0.01
	Lower arm	1.23	1.24	1.24	**1.24**	0.01
	Chest	1.06	1.07	1.05	**1.06**	0.01
	Front hip	1.07	1.12	1.07	**1.09**	0.02
	Back	1.07	1.06	1.07	**1.07**	0.01
	Back hip	1.15	1.13	1.15	**1.14**	0.01
Shirt loose	Upper arm	1.27	1.32	1.30	**1.30**	0.02
	Lower arm	1.27	1.29	1.30	**1.29**	0.01
	Chest	1.08	1.10	1.12	**1.10**	0.02
	Front hip	1.21	1.22	1.21	**1.21**	0.01
	Back	1.12	1.11	1.11	**1.11**	0.01
	Back hip	1.21	1.21	1.22	**1.21**	0.01
Smart shirt tight	Upper arm	1.36	1.37	1.38	**1.37**	0.01
	Lower arm	1.54	1.54	1.54	**1.54**	0.00
	Chest	1.11	1.10	1.12	**1.11**	0.01
	Front hip	1.04	1.04	1.05	**1.04**	0.00
	Back	1.07	1.08	1.07	**1.07**	0.00
	Back hip	1.09	1.08	1.09	**1.08**	0.00
Smart shirt regular	Upper arm	1.44	1.45	1.48	**1.46**	0.02
	Lower arm	1.57	1.57	1.56	**1.57**	0.00
	Chest	1.17	1.15	1.18	**1.17**	0.01
	Front hip	1.15	1.16	1.13	**1.15**	0.01
	Back	1.14	1.12	1.13	**1.13**	0.01
	Back hip	1.22	1.22	1.24	**1.23**	0.01
Smart shirt loose	Upper arm	1.54	1.51	1.58	**1.54**	0.03
	Lower arm	1.73	1.72	1.76	**1.74**	0.02
	Chest	1.18	1.20	1.20	**1.19**	0.01
	Front hip	1.33	1.38	1.38	**1.36**	0.02
	Back	1.19	1.17	1.17	**1.17**	0.01
	Back hip	1.34	1.27	1.26	**1.29**	0.04

Table S3 Local area factors mesured on manikin James – Part 2

Item	Body part	Local area factor James				
		1	2	3	avg	SD
Sweater	Upper arm	1.33	1.28	1.29	**1.30**	0.02
	Lower arm	1.36	1.33	1.33	**1.34**	0.01
	Chest	1.12	1.22	1.17	**1.17**	0.04
	Front hip	1.24	1.27	1.39	**1.30**	0.06
	Back	1.13	1.10	1.12	**1.12**	0.01
	Back hip	1.44	1.44	1.42	**1.43**	0.01
Jacket	Upper arm	1.79	1.63	1.61	**1.68**	0.08
	Lower arm	1.68	1.69	1.68	**1.69**	0.00
	Chest	1.44	1.31	1.31	**1.35**	0.06
	Front hip	1.54	1.31	1.37	**1.41**	0.09
	Back	1.14	1.16	1.12	**1.14**	0.02
	Back hip	1.19	1.41	1.38	**1.33**	0.10
Jeans tight	Front hip	1.07	1.06	1.06	**1.06**	0.00
	Back hip	1.00	1.00	1.00	**1.00**	0.00
	upper leg total	1.06	1.06	1.06	**1.06**	0.00
	lower leg total	1.26	1.27	1.28	**1.27**	0.01
Jeans regular	Front hip	1.07	1.06	1.07	**1.07**	0.01
	Back hip	1.01	1.00	1.00	**1.01**	0.00
	upper leg total	1.13	1.13	1.13	**1.13**	0.00
	lower leg total	1.41	1.41	1.41	**1.41**	0.00
Jeans loose	Front hip	1.11	1.11	1.11	**1.11**	0.00
	Back hip	1.02	1.02	1.01	**1.02**	0.00
	upper leg total	1.21	1.20	1.20	**1.20**	0.00
	lower leg total	1.56	1.55	1.56	**1.56**	0.00
Trousers	Front hip	1.07	1.06	1.08	**1.07**	0.01
	Back hip	1.05	1.04	1.04	**1.04**	0.00
	upper leg total	1.30	1.27	1.28	**1.28**	0.01
	lower leg total	1.57	1.56	1.56	**1.56**	0.00
Skirt	Front hip	1.35	1.30		**1.32**	0.03
	Back hip	1.22	1.16		**1.19**	0.03
	upper leg total	2.30	2.29		**2.29**	0.00

Table S4 Correction factors for total insulation of nude $I_{T,0.4ms,st}/I_{T.0.2ms,st}$ and clothing insulation $I_{cl,0.4ms,st}/I_{cl.0.2ms,st}$ of measured outfits

	Upper arm	Lower arm	Chest	Back	Front hip	Back hip	Upper leg	Lower leg
Nude	0.83	0.85	0.79	0.95	0.78	0.91	0.84	0.87
1	0.98		0.98	0.92	1.06	1.19	0.86	1.00
3	0.96	0.95	0.91	0.95	0.83	0.92	0.92	0.99
14	0.97	1.01	0.88	0.89	0.97	0.95	0.93	0.98
19	0.90	0.96	0.97	1.03	0.74	0.79	0.98	1.07
21	0.99	0.95	0.99	1.14	0.91	1.13	1.00	1.02
22	1.00	1.01	0.90	0.97	0.96	0.99	0.85	0.67

Table S5 Correction factors for total insulation of nude $I_{T,1ms,st}/I_{T.0.2ms,st}$ and clothing insulation $I_{cl,1ms,st}/I_{cl.0.2ms,st}$ of measured outfits

	Upper arm	Lower arm	Chest	Back	Front hip	Back hip	Upper leg	Lower leg
Nude	0.53	0.56	0.52	0.66	0.53	0.68	0.55	0.62
1	0.88	/	0.85	0.97	0.73	1.19	0.91	1.05
3	0.73	0.72	0.68	0.81	0.60	0.99	0.88	0.95
14	0.82	0.96	0.71	0.79	0.78	1.01	1.02	1.06
19	0.87	0.61	0.89	1.02	0.59	0.95	0.90	1.04
21	0.85	0.88	0.72	1.03	0.50	0.94	0.89	0.99
22	0.81	0.80	0.68	0.87	0.77	0.94	0.72	0.48

Table S6 Correction factors for total insulation of nude $I_{T,0.2ms,move}/I_{T.0.2ms,stand}$ and clothing insulation $I_{cl,0.2ms,move}/I_{cl.0.2ms,stand}$ of measured outfits

	Upper arm	Lower arm	Chest	Back	Front hip	Back hip	Upper leg	Lower leg
Nude	0.96	0.83	1.00	1.14	0.98	1.02	0.91	0.75
1	0.76		0.92	1.23	0.97	0.88	0.68	0.75
3	0.73	0.82	0.73	1.22	0.73	0.80	0.72	0.77
14	0.61	0.76	0.73	1.05	0.77	0.98	0.47	0.59
19	0.77	0.84	0.94	1.30	0.92	1.06	0.87	0.80
21	0.61	0.55	0.76	1.16	0.65	0.74	0.34	0.53
22	0.66	0.77	0.69	1.03	1.00	1.09	0.52	0.57

Table S7 Correction factors for total insulation of nude $I_{T,1ms,move}/I_{T.0.2ms,stand}$ and clothing insulation $I_{cl,1ms,move}/I_{cl.0.2ms,stand}$ of measured outfits

	Upper arm	Lower arm	Chest	Back	Front hip	Back hip	Upper leg	Lower leg
Nude	0.54	0.58	0.52	0.70	0.54	0.65	0.55	0.62
1	0.65		0.77	0.96	0.63	1.07	0.63	0.77
3	0.61	0.69	0.55	0.92	0.49	0.85	0.73	0.76
14	0.57	0.71	0.55	0.87	0.57	0.76	0.39	0.56
19	0.68	0.65	0.76	0.93	0.60	0.79	0.77	0.81
21	0.56	0.55	0.63	1.06	0.48	0.81	0.36	0.53
22	0.62	0.71	0.57	0.97	0.78	1.08	0.49	0.67

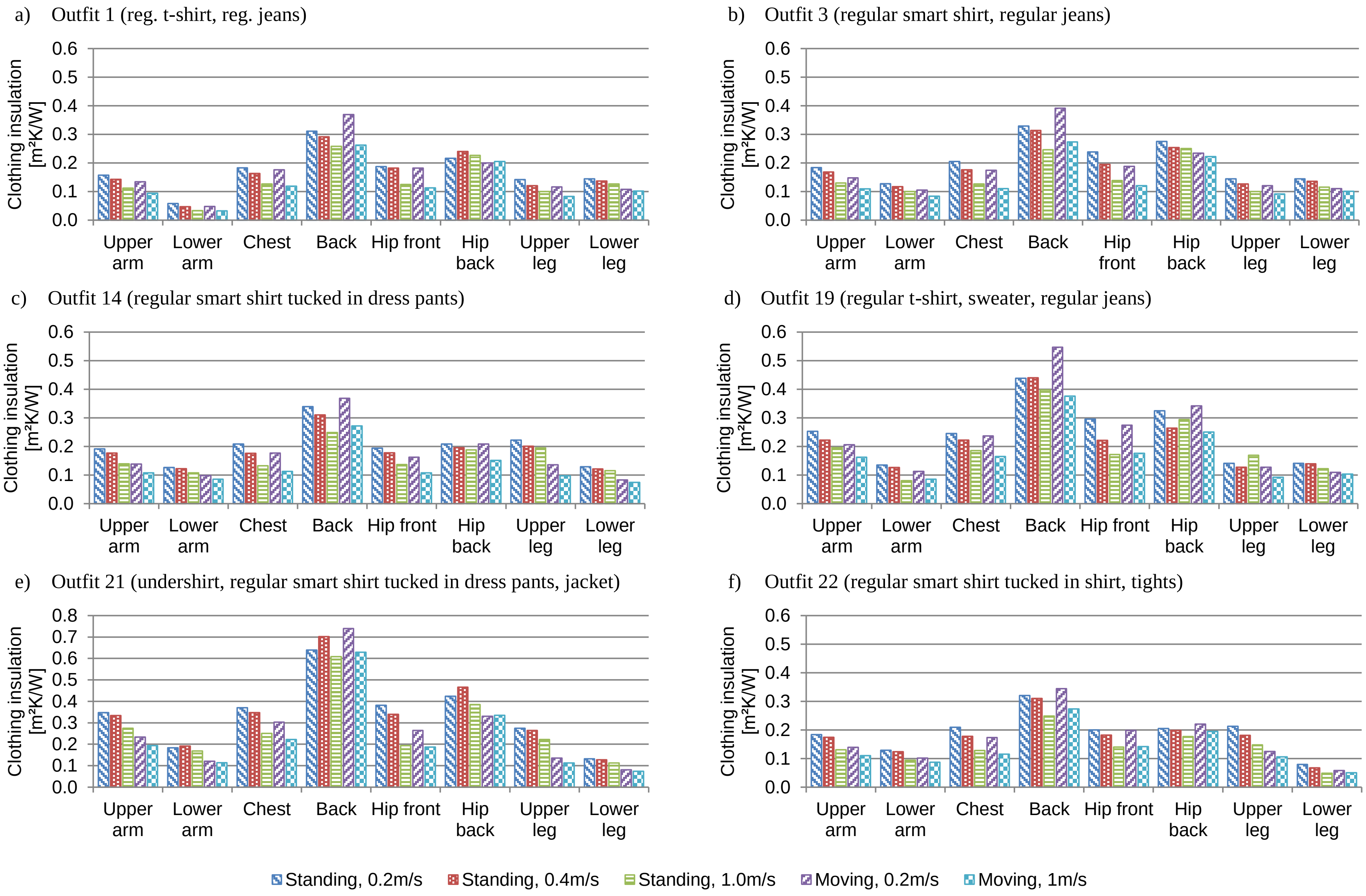

Figure S1 Influence of air speed and body movement on local total clothing insulation
